# Shift Current with Gaussian Basis Sets and General Prescription for Maximally Symmetric Summations in the Irreducible Brillouin Zone

**DOI:** 10.1021/acs.jctc.3c00917

**Published:** 2023-12-14

**Authors:** M. A. García-Blázquez, J. J. Esteve-Paredes, A. J. Uría-Álvarez, J. J. Palacios

**Affiliations:** † Departamento de Física de la Materia Condensada, Universidad Autónoma de Madrid, E-28049 Madrid, Spain; ‡ Condensed Matter Physics Center (IFIMAC), Universidad Autónoma de Madrid, E-28049 Madrid, Spain

## Abstract

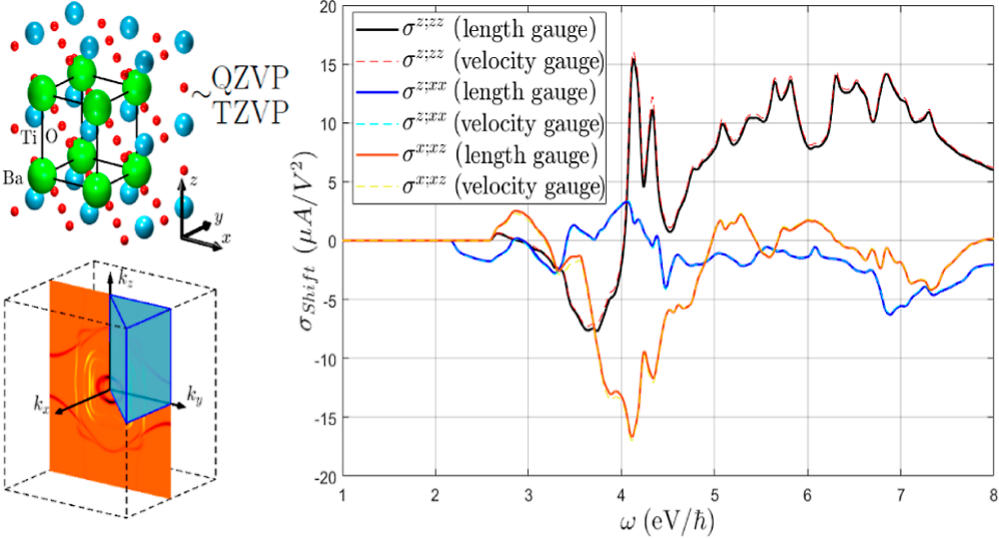

The bulk photovoltaic effect is an experimentally verified phenomenon by which a direct charge current is induced within a non-centrosymmetric material by light illumination. Calculations of its intrinsic contribution, the shift current, are nowadays amenable from first-principles employing plane-wave bases. In this work, we present a general method for evaluating the shift conductivity in the framework of localized Gaussian basis sets that can be employed in both the length and velocity gauges, carrying the idiosyncrasies of the quantum-chemistry approach. The (possibly magnetic) symmetry of the system is exploited in order to fold the reciprocal space summations to the representation domain, allowing us to reduce computation time and unveiling the complete symmetry properties of the conductivity tensor under general light polarization.

## 1 Introduction

The generation of a non-oscillating response in a material medium under an incident electric oscillating field is a general feature that occurs at all even orders in the perturbative expansion, that is, it is a nonlinear optical phenomenon. For responses that transform as vectors, such as an electric current, an elemental symmetry analysis shows that all the even order response tensors must vanish in the presence of inversion symmetry; hence, such frequency-independent quantities can only arise in non-centrosymmetric materials. In this regard, the emergence of a direct charge current in a homogeneous material induced by light is known as the bulk photovoltaic effect (BPVE).^1^ It was established by the mid-seventies with earlier experimental reports in ferroelectric materials,^2−5^ and continued gathering attention during the next decades.^6−14^ However, the potential applications in solar cells^15,16^ and the advances in both experimental facilities and first-principles capabilities have driven a considerable surge of studies in recent years.^17−26^

The BPVE is part of the total second-order optical response, which in addition includes second harmonics and, for polychromatic electric fields, contributions of mixed frequencies. In turn, the BPVE can be separated into 3 essentially different contributions:^17^ the shift current, a static and coherent (stemming from the off-diagonal part of the density matrix) response that under time-reversal  symmetry appears only with linearly polarized light; and two transient contributions that eventually reach a steady-state, namely the injection current, which under  symmetry appears only with circularly polarized light, and the ballistic current, which under  symmetry emerges purely from coherent scattering processes such as electron–phonon or electron–hole interactions that introduce an imbalance between the carrier generation rates across the Brillouin zone (BZ). Disregarding excitonic effects, the BPVE in nonmetallic systems occurs at frequencies above the band gap. These quantities, or equivalently the corresponding third-rank tensors σ^*a*;*bc*^(ω) as a function of a single frequency, admit expressions in terms of the quasiparticle properties that are amenable to numerical evaluation via quantum mechanical methods. Specifically, these microscopic expressions can be obtained by diagrammatic approaches for the ballistic current;^27−29^ and by solving the density matrix perturbatively,^1^ employing Wilson loops^30^ or again by diagrammatic techniques^31^ for the injection and shift currents. However, only the latter one is truly intrinsic to the single-particle system, in the sense that it can be computed exclusively from the band structure and electronic eigenfunctions without further modeling.

The calculation of the shift current presents some difficulties or subtleties starting from the choice of gauge for the interaction of electrons with the field.^31−34^ The most generally applicable method, the length gauge, requires evaluation of numerical derivatives with respect to the crystalline momentum ***k*** of quantities that are not gauge invariant. In contrast, the velocity gauge constitutes a more straightforward alternative, although it carries an (a priori) infinite sum over the electronic states external to the direct optical transition. Both gauges require the evaluation of the matrix representation of the velocity operator in the set of crystalline eigenfunctions, and both are expected to yield equal results in the limit of a complete basis for describing the latter. There currently exist methods for evaluating the shift current in the single-particle approximation from density-functional theory (DFT),^35^ tight-binding (including Wannierizations^36,37^), and ***k***·***p*** band structures.^18^ Yet, as is often the case in physics-leaning studies, the DFT calculations are almost invariably assumed to employ a plane-wave basis, at least when the velocity operator is not approximated by the momentum.

In this work, we present a formalism for computing the shift conductivity tensor in nonmetallic crystals, in both length and velocity gauges, from first-principles employing Gaussian basis sets. It is based on an exact calculation of the velocity and Berry connection matrix elements through an analytical evaluation of the real-space integrals involved. The use of a localized basis presents some advantages and disadvantages with respect to the plane-wave alternative inherited from the DFT methods: the whole chain of calculations should generally be faster, the evaluation of position (and by extension, velocity) matrix elements is straightforward, hybrid functionals can be used at a low cost (which may allow an accurate gap to be obtained avoiding scissor corrections or GW calculations), all-electron calculations can be performed, and no artificial replication of layers is required in 2D materials. On the other hand, a customized basis optimization has to be performed for each system while limited by the superposition error and diffusive exponents, and errors from the lack of completeness of the basis are more likely (making the more delocalized unoccupied states particularly hard to reproduce). It is expected that the reliability of this method is highly correlated to its ability to properly reproduce the relevant occupied and unoccupied states (dictated by the frequency range) with a Gaussian basis.

A further benefit of the use of localized bases lies in the guarantee that the complete symmetry of the system is preserved, in slight contrast with the maximally-localized Wannier representation. The properties of the crystallographic point group can then be exploited to reduce the summations over the BZ that are required for the shift conductivity to properly weighted sums, which encode the whole (possibly magnetic) symmetry of the system, only over its irreducible part or representation domain. We present a complete list of the explicit formulas for each space group, including the magnetic configurations, where the structure type is used to parametrize the irreducible domain and the (magnetic) point group determines the precise folding of the ***k***—resolved conductivity.

## 2 Shift Current: Definition, Considerations, and Numerical Evaluation

A general expression for the total second-order optical response under homogeneous illumination can be obtained by solving the density matrix in perturbation theory for the field. In particular, for a uniform polychromatic electric field , the shift current is defined as the intrinsic second-order DC component
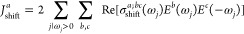
1where *a*, *b*, and *c* label the spatial components in the chosen coordinate system.

### 2.1 Length Gauge

In the length gauge, the electric potential is chosen as  and the perturbative expression for the shift conductivity third-rank tensor in a nonmetallic material ultimately reads^14,21,38^
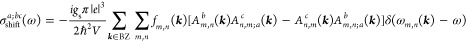
2which is valid irrespective of whether time-reversal  is a symmetry. In this formula,

- *m* and *n* label the eigenstates of the periodic single-particle Hamiltonian , which satisfy Bloch’s theorem: ⟨***r***∥ψ_*n*,***k***_⟩ = ψ_*n*,***k***_(***r***) = *e*^*i****kr***^*u*_*n*,***k***_(***r***) with *u*_*n*,***k***_ having the periodicity of the direct lattice. ***k*** is the crystalline momentum or label of the irreducible representations of the translation group. *ℏ*ω_*m*,*n*_(***k***) ≡ *E*_*m*,***k***_ – *E*_*n*,***k***_ and *f*_*m*,*n*_(***k***) ≡ *f*_*m*_(***k***) – *f*_*n*_(***k***) is the difference of Fermi distributions, hereafter taken at zero temperature.
is the *b*th spatial component of the Berry connection matrix elements, which satisfy *A*_*m*,*n*_^*b*^(***k***) = *A*_*n*,*m*_^*b*^(***k***)*.
- *A*_*n*,*m*;*a*_^*b*^(***k***) = ∂_*k*′_^*a*^*A*_*n*,*m*_^*b*^(***k***′)|_***k***_ – *i*[*A*_*n*,*n*_^*a*^(***k***) – *A*_*m*,*m*_^*a*^(***k***)]*A*_*n*,*m*_^*b*^(***k***) is the generalized derivative (GD) of the Berry connection.
- *V* = *N*_***k***_*V*_PUC_ is the volume (area in 2D, or longitude in 1D) of the crystal, with *N*_***k***_ → ∞, the number of terms in the BZ summation (or discretized integration), and *V*_PUC_, the volume of the primitive unit cell. δ(ω) is a nascent (or broadened) Dirac delta function. *g*_s_ = 1 (2) in the presence (absence, respectively) of spin-dependent terms in the Hamiltonian (excluding the doubled states in the *m* and *n* summations).
- We have introduced a global minus sign in agreement with ref (21) and eq 58 of ref (14). We note that some authors do not include the 1/2 factor in the conductivity, instead canceling it with the prefactor in (1).

It follows that in general, σ_shift_^*a*;*bc*^(ω) = σ_shift_^*a*;*cb*^(ω)* = σ_shift_^*a*;*bc*^(−ω)* and σ_shift_^*a*;*bb*^ is real. For linearly polarized light, *E*^*b*^(ω_*j*_)*E*^*c*^(−ω_*j*_) is real for all components and only Re σ_shift_^a;bc^ contributes to *J*_shift_^*a*^. In contrast, for circular polarization, *E*^*b*^(ω_*j*_)*E*^*c*^(−ω_*j*_) is complex for some *b* and *c*, hence both the real and imaginary parts of the conductivity may contribute to the current in general. Under  symmetry, in particular excluding any permanent magnetic alignment,  for an arbitrary phase θ_*n*,***k***_; hence, applying the antiunitary transformation in the inner products and noting that  we obtain
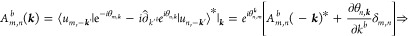

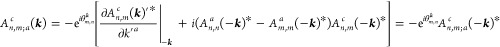
where θ_*n*,*m*_^***k***^ ≡ θ_*n*,***k***_ – θ_*m*,***k***_. Thus, *f*_*m*,*n*_(***k***)*A*_*m*,*n*_^*b*^(***k***)*A*_*n*,*m*;*a*_^*c*^(***k***) = −[*f*_*m*,*n*_(−***k***)*A*_*m*,*n*_^*b*^(−***k***)*A*_*n*,*m*;*a*_^*c*^(−***k***)]*, and σ_shift_^*a*;*bc*^ is real with  symmetry. In this case also, −*A*_*n*,*m*_^*c*^(***k***)*A*_*m*,*n*;*a*_^*b*^(***k***) = *A*_*m*,*n*_^*c*^(***k***)*A*_*n*,*m*;*a*_^*b*^(***k***) under the BZ summation. The difference between the currents for right and left circular polarization, which is proportional to Im σ_shift_^*a*;*bc*^ only, is therefore vanishing; and the shift current is associated with linearly polarized light. In the event of  breaking, a circular shift current generally emerges as well as a nonstationary injection current.^21,23^ We remark that the injection current can be equally computed with the method described in this work, but we do not show explicit results since the calculation is straightforward compared to that for the shift current,^14,38^ up to an extrinsic scattering rate that is often set phenomenologically.

### 2.2 Berry Connection and Velocity in a Local Basis

The single-particle crystalline eigenstates are generally expanded in a set of states^a^ satisfying Bloch’s theorem as
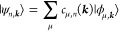
where μ is, in principle, a generic label and the coefficients are obtained from the generalized eigenvalue problem
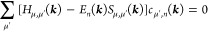
where the Hamiltonian *H*_μ,μ′_(***k***) and overlap *S*_μ,μ′_(***k***) matrix elements are the representations of the Hamiltonian *Ĥ* and identity *Î* operator, respectively, in the set {|ϕ_μ,***k***_⟩}_μ_ for each ***k***. In a local basis, which is repeated in each unit cell and labeled by the lattice vectors ***R***, the Bloch states can in turn be expanded in agreement with Bloch’s theorem:
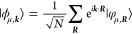
where *N* = *V*/*V*_PUC_ → ∞ is the number of unit cells in the crystal. Therefore, due to the periodicity of *Ĥ*(*r*) and *Î*,
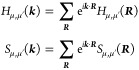
3where *H*_μ,μ′_(***R***) = ⟨φ_μ,**0**_|*Ĥ*|φ_μ′,***R***_⟩ and *S*_μ,μ′_(***R***) = ⟨φ_μ,**0**_|φ_μ′,***R***_⟩.

The Berry connection matrix elements can then be expressed in the local basis by inserting the transformation , yielding after some algebra
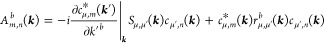
which can be compactly expressed in matrix form as
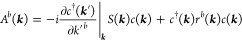
4We have introduced the position matrix elements in the Bloch basis,
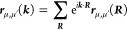
5with
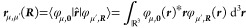
which is well-defined in a localized basis, albeit the diagonal components depend on the origin choice. Indeed, a rigid shift of the form ***r*** → ***r*** + ***r***_0_ (which does not alter the position operator ***r̂*** itself) with ***r***_0_ restricted to the unit cell results in *A*_*m*,*n*_^*b*^(***k***) → *A*_*m*,*n*_^*b*^(***k***) – ***r***_0_δ_*m*,*n*_. Nevertheless, it is easy to see that this arbitrary factor is canceled in the GD *A*_*m*,*n*;*a*_^*b*^, rendering the shift conductivity invariant under this choice.

As can be observed in eq 4, the calculation of both diagonal and off-diagonal Berry connections requires evaluating numerical derivatives with respect to ***k***. In order to avoid further complications in the term ∂_*k*_^*a*^*A*_*n*,*m*_^*b*^ of the GD *A*_*n*,*m*;*a*_^*b*^, such as the introduction of a second grid for derivatives or the increase in the required significant digits, we employ the identity
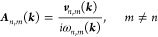
6which can be readily obtained by expanding ∇_***k***_[⟨*u*_*m*,***k***_|e^–*i****k***·***r***^*Ĥ*e^*i***k**·***r***^|*u*_*n*,***k***_⟩] = 0 for *m* ≠ *n*. In eq 6, ***v***_*n*,*m*_(***k***) = ⟨ψ_*n*,***k***_|***v̂***|ψ_*m*,***k***_⟩ = ***v***_*m*,*n*_(***k***)* are the matrix elements (here in the set of eigenstates) of the velocity operator ***v̂*** ≡ . It can be shown that the velocity matrix elements have the following form in the local basis:^39^
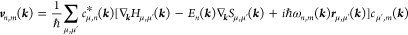
7which is independent of the origin choice. The ***k*** derivatives in this expression are all analytical, in particular ∇_***k***_*H*_μ,μ′_(***k***) = *i∑*_***R***_e^*i****k***·***R***^***R****H*_μ,μ′_(***R***) and likewise for *S*_μ,μ′_(***k***). Therefore, ∂_*k*_^*a*^*A*_*n*,*m*_^*b*^ and *A*_*n*,*m*_^*b*^ can be computed employing eq 6 with numerical derivatives only of the first and zeroth order, respectively. Inserting these into eq 2, noting that the *f*_*m*,*n*_ factor forces *m* ≠ *n* and that the derivatives of ω_*n*,*m*_ cancel out, one obtains
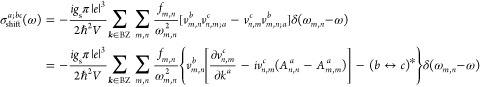
8where (*b* ↔ *c*)* represents the complex conjugate of the previous term inside the curly brackets with the components *b* and *c* permuted (even if *b* = *c*), and the ***k*** dependence has been omitted for brevity. Note that with  symmetry, the – (*b* ↔ *c*)* term is equivalent to + (*b* ↔ *c*) under the BZ summation.

A subtle issue in (8) and other equivalent length gauge formulas is the evaluation of numerical derivatives of quantities that are not gauge-invariant, in particular the coefficients *c*_μ,*m*_ in the diagonal Berry connections, see (4), and the velocities ***v***_*n*,*m*_ for *n* ≠ *m*; which are respectively determined up to arbitrary phase factors θ_*m*,***k***_ and θ_*m*,*n*_^***k***^ = θ_*m*,***k***_ – θ_*n*,***k***_. The continuity of (3) in ***k*** makes all *c*(***k***) (and ***v***(***k***) by extension) also continuous, except in general for the phases. In order to fix a continuous gauge, we impose that
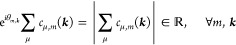
The necessary ***k*** derivatives are well-defined this way, and the θ_*m*,***k***_ factors are all canceled in the gauge-invariant eq 8 by a similar argument than in the time-reversal case above. The only remaining caveat is to keep track of the correct band indexing when a degeneracy occurs between the infinitesimally close ***k*** ± ***h*** points defining the numerical derivatives. However, this issue may be neglected by mapping the ***k*** summation to the interior of the irreducible BZ (IBZ), where only accidental degeneracy may occur (see Section 3).

In 2D materials, the out-of-plane tensor components, i.e., involving at least one index along the nonperiodic direction ***z***, can be computed on equal footing (and likewise for 1D systems). Regarding the system as a periodic stacking of layers, *H*_μ,μ′_(***R***_*z*_)^b^, *S*_μ,μ′_(***R***_*z*_), and ***r***_μ,μ′_(***R***_*z*_) are exponentially vanishing for interlayer vectors ***R***_*z*_ in the limit of large separation, making all *k*^*z*^ derivatives null. In this case, eqs 4 and 7 reduce to . While the *a* = *z* component in the shift conductivity tensor may not be of interest, in some point groups the IBZ summation requires the calculation of some of these components for *a* = *x*, *y*; in which case, the numerical derivatives in eq 8 are canceled and the expression is simplified significantly.

It may be worth noting that eqs 2 and 8 are often cast in terms of the shift vector^1,14,21^*R*_*n*,*m*_^*c*;*a*^ = *i*∂_*k*_^*a*^ log *v*_*n*,*m*_^*c*^ + *A*_*n*,*n*_^*a*^ – *A*_*m*,*m*_^*a*^ by noting that in the GD ∂_*k*_^*a*^*A*_*n*,*m*_^*b*^ = *A*_*n*,*m*_^*b*^∂_*k*_^*a*^ log *A*_*n*,*m*_^*b*^ when *A*_*n*,*m*_^*b*^ ≠ 0. The phase of the velocity can also be introduced explicitly through ∂_***k***_^***a***^Φ_*n*,*m*_^*b*^(***k***), where , yielding the term inside the curly brackets in eq 8 equivalent to
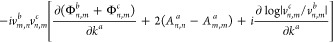
For linearly polarized light one can always rotate the coordinate system, initially based on the crystallographic structure, such that ***E***(ω) points along, say, the *b* direction. Then only the σ^*a*;*bb*^ component contributes to the current along *a* in eq 1 and, in particular, the modulus term vanishes in the last expression. Regardless, there are approaches to compute the shift vector that do not explicitly involve the derivative of the phase.^30,35^ In this work, eq 8 is employed directly.

### 2.3 Evaluation in Gaussian Basis Sets

The evaluation of eq 8 from first-principles requires thus the knowledge of the matrix elements of *Ĥ*, *Î*, and ***r*^** in the local basis for a sufficiently large number of lattice vectors. The first one, *H*_μ,μ′_(***R***), must be evaluated self-consistently, typically in a DFT or hybrid DFT-HF (Hartree–Fock) scheme; and is generally expected to be provided by the corresponding electronic structure code for the chosen functional. The latter two, *S*_μ,μ′_(***R***) and ***r***_μ,μ′_(***R***), can be manually precomputed from the (possibly optimized) atomic structure. If the local functions are harmonic Gaussian-type orbitals (GTOs), this can be done analytically. In that case, μ is a multi-index labeling the atoms *a* (located at ***d***_*a*_) in the unit cell^c^, the pair of orbital quantum numbers *l*, *m* (*m* = −*l*, ..., *l*), the shells λ discerning the harmonics with identical *l* and, in the presence of spin-dependent terms in *Ĥ* such as spin–orbit coupling (SOC) or magnetic ordering, the *m*_s_ = ±1/2 spin quantum number. The contracted real GTOs  are then defined as^41,42^
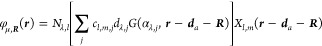
where *N*_λ,*l*_ and *c*_*l*,*m*,*j*_ are normalization coefficients (see Appendix E of ref (41)), *d*_λ,*j*_ and α_λ,*j*_ are the selected contraction coefficients and exponents,  are the Gaussian-type radial functions ,and *X*_*l*,*m*_(***r***) are the real solid harmonics. The latter are obtained from the (not normalized) spherical harmonics *Y*_*l*,*m*_(***r***) as
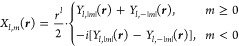
The central integrals that need to be evaluated to obtain *S*(***k***) and ***r***(***k***) are then
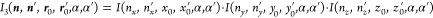
where
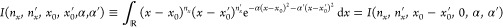
9with ***n*** = (*n*_*x*_, *n*_*y*_, *n*_*z*_) and ***n***′ = (*n*_*x*_′, *n*_*y*_′, *n*_*z*_′) determined by the *l*, *m* quantum numbers of the bra and ket GTOs, respectively. The 1D integrals appearing in ***r***(***k***) are then computed as
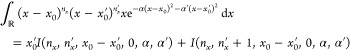
There are several methods to tabulate the 1D integrals in eq 9, e.g., by recursion over *n* and *n*′. In this work, we have instead employed the following master expression, which can be deduced from ref (43):
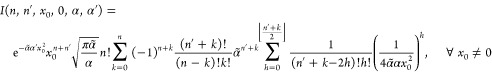
where  and
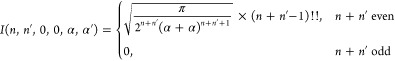
We note that only one of *S*_μ,μ′_(±***R***) (and likewise for ***r***_μ,μ′_(±***R***)) needs be computed for each ***R*** ≠ **0** since
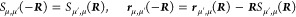
and only the upper or lower triangle for ***R*** = **0**. If needed, the number of matrix elements may be further restricted such that only atoms in the asymmetric unit are considered in, e.g., the bra. The remaining entries can then be reconstructed by employing the (spinless) transformation properties of the real solid harmonics, *ĝ*^–1^*X*_*l*,*m*_(***r***) = *X*_*l*,*m*_(*g**r***) = , and the position operator, , where *g* ∈ *O*(3) and  is the representation of *O*(3) of angular momentum *l*(44) (*l* = 1 for ***r***). The result is
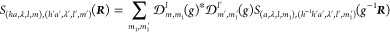

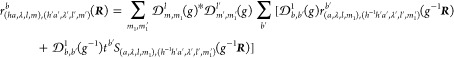
10where *h* = (*g*|***t***) is a general nonsymmorphic operation in the crystallographic point group *F* (or space group excluding lattice translations, *G*/*T*) and *ha* represents the atom located at *h****d***_*a*_. Note that the atom *h*^–1^*h*′*a*′ may require a nontrivial lattice vector in order to be mapped to the unit cell, thus altering *g*^–1^***R***. If SOC is considered, then , where  (in the {↑, ↓} basis order) is the projective representation of *O*(3) of angular momentum 1/2, which is even under inversion *i*; σ is the Pauli vector, and ***e*** is the counterclockwise rotation axis. The previous relations would be modified in consequence.

In plane-wave schemes, ***v̂*** is sometimes replaced by the momentum . This substitution is not exact in HF or hybrid DFT-HF schemes because ***r̂*** and the Fock operator do not commute, or likewise when employing nonlocal pseudopotentials (PP) or including relativistic effects such as SOC. While the deviations in the final quantities are often not large,^37^ the increase in computational cost in the Gaussian scheme is marginal enough to advise against the use of this approximation in general, except perhaps for extremely large unit cells. Regardless, the relevant integrals would be computed as
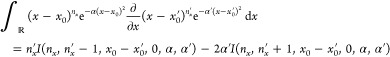


### 2.4 Velocity Gauge

Alternatively, the velocity gauge can be employed by imposing the minimal coupling , where  is the vector potential. An analogous derivation in perturbation theory then yields^8,23,31,33^
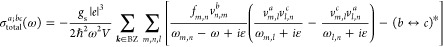
Both the shift and the injection conductivities are encoded in this formula. The former can be obtained by taking only the imaginary part of the product of complex denominators in the ε → 0^+^ limit, which is equivalent to taking Re σ_total_^a;bc^ under  symmetry (i.e., considering linear polarization)
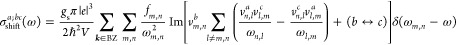
11In contrast with eqs 2 and 8, the evaluation of eq 11 avoids the numerical derivatives at the cost of a sum over all states *l* that are external to the direct optical transition. Resulting from the completeness relation , in principle it must span the whole set of bands (which with localized bases is seldom demanding, computationally) even if they are not well represented above a certain window from the Fermi level, but the sum should nevertheless converge to the correct result when a sufficiently large basis is employed. While the length gauge explicitly involves only the pair of bands corresponding to the direct optical transition at the field frequency, a large basis should still be needed to properly reproduce the conduction bands involved. For grids of equal size, the evaluation of eq 11 is more straightforward and less computationally demanding than eq 8; however, the assumption of completeness (which is not strictly true in finite bases) makes the length gauge approach the most reliable in general.

A similar expression to eq 11 that is frequently employed in the literature is obtained within the length gauge by employing the following sum rule for the GD:
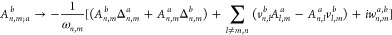
12where  and **Δ**_*n*,*m*_(***k***) ≡ ***v***_*n*,*n*_(***k***) – ***v***_*m*,*m*_(***k***) = ∇_*k*_ω_*n*,*m*_(***k***).^d^ This sum rule can be obtained by expanding  and inserting the completeness relation above. Employing eq 12 in eq 2 yields
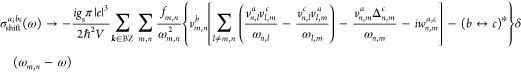
13where we note that the Δ^*a*^ terms cancel out. Clearly, this expression coincides with eq 11 in the presence of  symmetry, except for the last two terms inside the square brackets. The term *w*^*a*,*c*^, which would clearly vanish in the absence of nonlocal terms in the Hamiltonian , is often computed in tight-binding or Wannier schemes by differentiating the Hamiltonian matrix,^18,37^ but its calculation in pure DFT is nontrivial and often ignored, leaving the two-band **Δ** terms as the only difference in practice between the velocity gauge (eq 11) and length gauge with sum rule (eq 13) expressions. While the contribution of these terms is usually small, in Section 2.5 we show that the proper agreement of the length gauge in eq 2 or 8 is with the velocity gauge expression (eq 11), at least if one neglects *w*^*a*,*c*^.

### 2.5 First-Principles Results and Discussion

**Figure 1:**
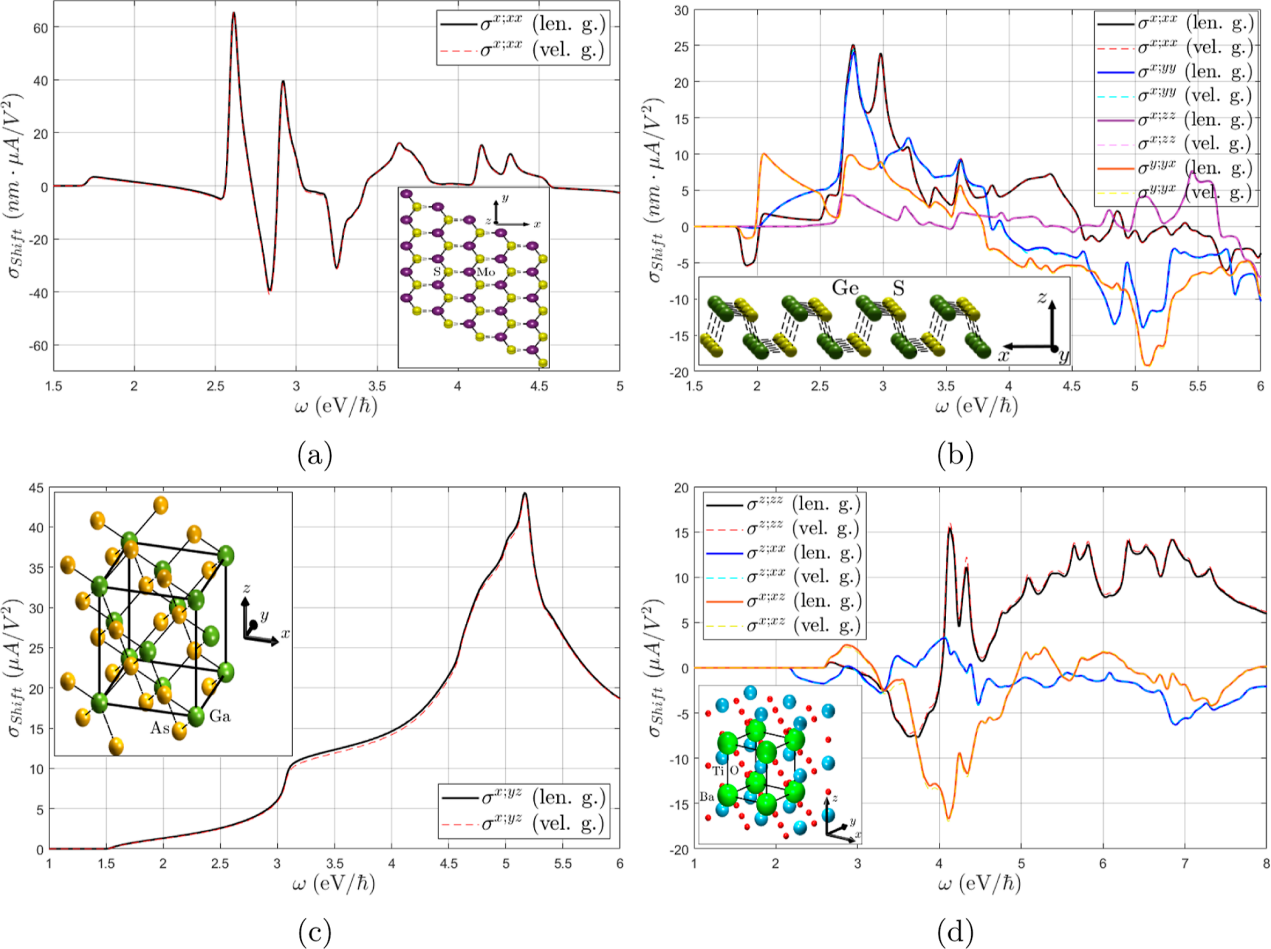
Shift conductivity tensor in (a) monolayer MoS_2_, (b) monolayer GeS, (c) GaAs, and (d) BaTiO_3_ computed in both the length (eq 8) (solid lines) and velocity gauge (eq 11) (dashed lines). All linearly independent components are shown in each case, excluding σ^*z*;*zx*^ in GeS for an out-of-plane current. IBZ summations with time-reversal symmetry (eq 19) have been employed.

In Figure 1, we show the shift conductivity computed with large-sized Gaussian basis sets for some representative nonmagnetic materials, in both the length (eq 8) and velocity (eq 11) gauges. The self-consistent electronic structure problem has been solved with the CRYSTAL code,^45,46^ from which the *H*_μ,μ′_(***R***) are readily obtained. The input files for the self-consistent calculations can be found in the Supporting Information, in addition to the resulting band structures.

The starting points for the basis sets were the following: in MoS_2_, def2-QZVP^47^ for S and pob-TZVP-rev2^48^ for Mo; in GeS, def2-QZVP; in GaAs, m-pVDZ-PP-Heyd;^49^ and in BaTiO_3_, def2-QZVP with PP from pob-TZVP-rev2^50^ for Ba. In all cases, the bases were modified in order to enable (or preserve) the convergence and obtain a sufficiently accurate band structure for the conduction bands in the energy ranges displayed in Figure 1, except for GaAs, which already presented a good dispersion with the unmodified Heyd basis. The standard GGA PBE functional^51^ was used in MoS_2_, GeS, and BaTiO_3_ in order to facilitate contrasting with the literature, while the short-range corrected hybrid HSE06 functional^52^ was employed in GaAs for the same reason. In the latter case, the use of a hybrid functional allows to obtain the experimental band gap at Γ of ∼1.5 eV, avoiding the use of a scissor correction or a *GW* calculation.

The initial grids for the conductivity contained 2000 × 2000 points in the BZ for MoS_2_, 1500 × 1500 for GeS, 400 × 400 × 400 for GaAs, and 200 × 200 × 200 for BaTiO_3_^e^; and were subsequently restricted to the IBZ as explained in Section 3, in particular employing eq 19 due to the absence of magnetism and the expressions from the list for the corresponding space groups: 187 (*D*_3*h*_) reduced to 2D for MoS_2_, 31 (*C*_2*v*_) reduced to 2D with *c*_2,*x*_ contained in the lattice plane for GeS, 216 (*T*_*d*_) for GaAs, and 99 (*C*_4*v*_) for BaTiO_3_. The delta function in the σ_shift_^*a*;*bc*^ expressions has been approximated by a narrow normal distribution  with a standard deviation σ = 20 meV in all cases. The absence of (unphysical) rapid fluctuations in the curves indicates that this value is not small for the chosen ***k*** grids. The numerical derivatives in the length gauge expression (eq 8) have been symmetrized as , with  in all cases. The direct lattice summations in eqs 3 and 5 have been truncated to the first (by length) 179 vectors in MoS_2_, 120 in GeS, 179 in GaAs, and 260 in BaTiO_3_.

The agreement with the results in the literature is generally good,^19,23,35−37,53^ specially taking into account that moderate discrepancies can be found commonly due to the high sensibility of σ_shift_ to the lattice parameters, atomic coordinates, and electronic eigenfunctions,^18,26,54^ in conjunction with the different calculation methods (as outlined in the previous sections) and convergence parameters such as the BZ grid or the broadening of the delta functions. The proper description of the eigenstates in the *ℏ*ω energy range is critical to obtaining satisfactory results; hence, in principle, the largest possible basis set allowed by convergence should be used, typically a reduced QZVP or augmented TZVP. Nevertheless, in materials with not particularly delocalized empty conduction states a smaller but properly calibrated basis could suffice. It should be kept in mind that the truncation of the ***R*** sums may need to be shortened if smaller Gaussian exponents are introduced.

**Figure 2:**
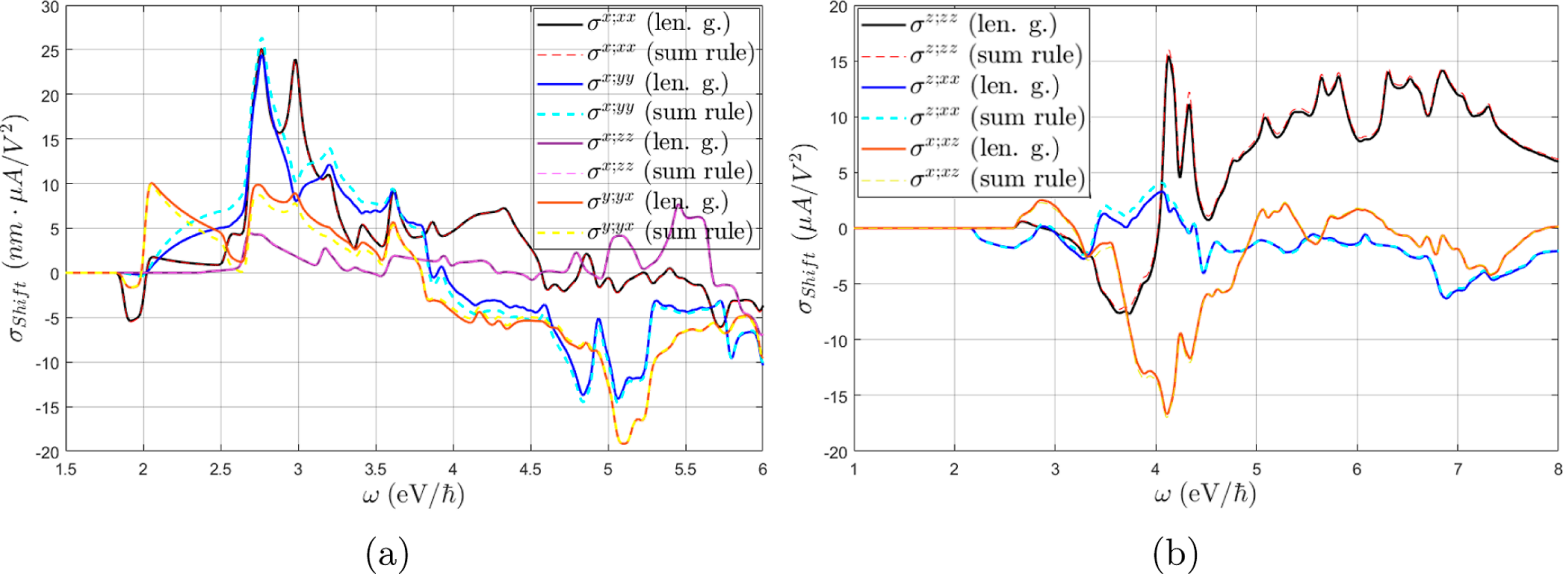
Shift conductivity tensor in (a) monolayer GeS and (b) BaTiO_3_ computed in the length gauge (eq 8) (solid lines) and employing the sum rule (eq 13) (dashed lines), excluding the *w*^*a*,*c*^ term. IBZ summations with time-reversal symmetry (eq 19) have been employed.

It can be observed in Figure 1 that the results of both length and velocity gauges are virtually identical in all cases, even if the external sum in eq 11 is truncated in practice by the finiteness of the basis. This is in agreement with the results of ref (34) for third-order calculations in graphene. However, as displayed in Figure 2, the inclusion of the two-band **Δ** terms in eq 13 by the sum rule induces a small but noticeable discrepancy in some of the σ_shift_^*a*;*bc*^ components, specifically σ^*x*;*yy*^, σ^*y*;*yx*^ in GeS, and σ^*z*;*xx*^ in BaTiO_3_, while all other components (including MoS_2_ and GaAs) were not visibly affected. Most of the unaltered components have a symmetry reason to remain so: the **Δ** terms clearly do not contribute to *a* = *b* = *c* components in eq 13, nor to *b* = *c* = *z* components in 2D materials since Δ^*z*^ = 0. Furthermore, it can also be seen from eq 13 that a sum over even permutations of (*a*, *b*, and *c*) cancels the **Δ** terms, which is precisely underlying in GaAs, as can be seen from the folded summation formula for *T*_d_ in Section 3. Only the σ^*x*;*xz*^ component in BaTiO_3_ cannot be explained by symmetry reasons, albeit in this case only a term of the form *v*_*n*,*m*_^*z*^*v*_*m*,*n*_^*x*^Δ_*n*,*m*_^*x*^ contributes under  symmetry, which is most likely small for numerical reasons in the dispersion along *k*^*x*^. It should be remarked that these discrepancies may possibly be reduced if the *w*^*a*,*c*^ term is not neglected (eq 13).

## 3 IBZ Summation

The proper use of GTOs ensures that the crystalline eigenstates indeed transform according to the irreducible representations of the space group *G*, and *ĥ*ψ_*n*,***k***_(***r***) = ψ_*n*,*g**k***_(***r*** – ***t*** – ***R***) = ψ_*n*,***k***_(*h*^–1^***r***) ∀*h* = (*g*|***t*** + ***R***) ∈ *G*. Then, since the velocity operator transforms under the corresponding change of coordinates as  (see discussion around eq 10 for context),^f^ and likewise for , the following identities must be satisfied, up to an arbitrary phase factor in the eigenstates that has no impact on the conductivity:
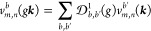

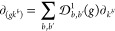

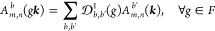
14where *F* is the (isogonal) point group of the material, formed by disregarding all translations of the space group. On the other hand, under the time-reversal operation  (odd power of time units), so that recalling from Section 2.1 the action of  on the Berry connection, if time-reversal is a symmetry then
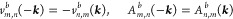
15where again the arbitrary phase factors have already been canceled. We note that eqs 14 and 15 hold irrespective of whether SOC is included, since the space group is the same^g^ and the representations of the eigenstates are not being used (but rather of the operators), and the sign of  has no impact. Consequently, the following analysis is also valid irrespective of SOC.

These transformation properties can now be exploited to reduce the summations over the BZ in eq 2, 8, or 13 to properly weighted sums over the IBZ or representation domain, from which the whole BZ is reconstructed by applying point group symmetries (possibly including ). This ensures that each sampled ***k*** point provides unique information, reducing the computation time of the original BZ grid by approximately the order of the point group and avoiding the touching of symmetry-enforced degeneracies, which are troublesome for computing the numerical derivatives in the length gauge. Furthermore, the identification of the finite tensor entries and the linear dependencies between them is straightforward from the IBZ expressions.

In order to avoid exceptions with duplicate points, we hereafter assume that the BZ sampling does not contain any high symmetry point (or, to be precise, any point whose little group is not trivial), which is without a loss of generality in sufficiently fine grids. Several cases are distinguished depending on time-reversal symmetry, which determines the magnetic point group *M*.

(*i*) *Magnetic point group of type I*: *M* contains only unitary operations, i.e., *M* = *F* and  is excluded. Then by eq 14, the first term in eq 8 satisfies
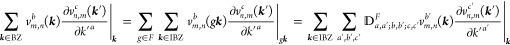
and likewise for the other terms, also in the form of eq 2 or 13. Here, we have defined, for any subset *L* ∈ *O*(3),
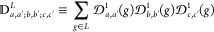
16Then, writing the ***k*** integrand explicitly for any of the forms eq 2, 8, or 13,
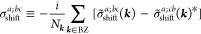
17it follows that
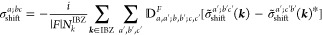
18where we have used the fact that *N*_***k***_ = |*F*|*N*_***k***_^IBZ^, where |*F*| is the order of *F* and *N*_***k***_^IBZ^ is the number of grid points in the IBZ. It is easy to see that  for any group with inversion symmetry *i* ∈ *F*, and hence σ_shift_^*a*;*bc*^ = 0, in agreement with the obvious requirement from perturbation theory.

(*ii*) *Magnetic point group of type II*: Time-reversal is a symmetry by itself, i.e., . This precludes any permanent magnetic ordering. Then by eqs 14 and 15,


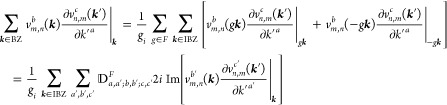
where *g*_*i*_ = 2 if *i* ∈ *F* or in the 2D cases with out-of-plane *c*_2,*z*_ rotational symmetry, and *g*_*i*_ = 1 otherwise. The group *F* is again forced to be non-centrosymmetric, but time-reversal symmetry effectively halves the IBZ with respect to the type I case by introducing a relation between the ± ***k*** pairs (except in the cases where *g*_*i*_ = 2). The general shift current tensor (eq 17) thus satisfies
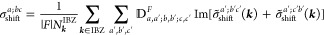
19where *N*_***k***_ = 2|*F*|*N*_***k***_^IBZ^/*g*_*i*_. In agreement with Section 2.1, σ_shift_^*a*;*bc*^ is real in this case.

(*iii*) *Magnetic point group of type III*: Exactly half of the unitary operations are paired with time-reversal, i.e., , where *H* ⊂ *F* is an ordinary point group and *F* – *H* is thus not a group. In this case, eq 14 is valid for *g* ∈ *H* whereas eq 15 must be used in combination with eq 14 for *g* ∈ *F* – *H*, and the IBZ is defined by *F* as in type I. Hence, symmetrizing in ±***k***,
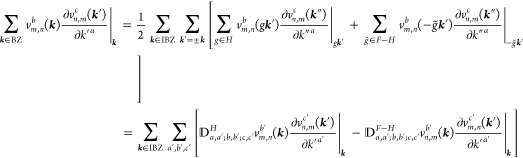
and, noting that , eq 17 can be expressed as
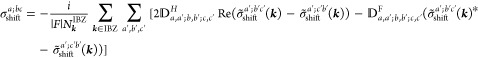
20If the crystalline structure is centrosymmetric disregarding magnetism, there are three cases upon magnetization. The first one is that *i* is completely removed by the magnetization, *i* ∉ *F* and *M* is of type I or III, thus it imposes no restrictions on σ_shift_^*a*;*bc*^. The second one is *i* ∈ *H* ⊂ *F*, which implies that  and σ_shift_^*a*;*bc*^ = 0. The third one is *i* ∈ *F* – *H*, which implies that  and Re σ_shift_^*a*;*bc*^ = 0. Therefore, a centrosymmetric material may host a finite shift current upon magnetization as long as inversion is not a symmetry by itself but in combination with time-reversal (often termed  symmetry), a situation that is most common in antiferromagnets and which, by eqs 1 and 20, yields a shift current under circular (or elliptical) polarization.

Note that eqs 18, 19, and 20 also include the case of 2D materials with the global symmetry σ_*h*_ ∈ *F* (mirror plane parallel to the lattice), since the required 1/2 factor in the *∑*_***k***∈BZ_ → (1/2)*∑*_*g*∈*F*_*∑*_***k***∈IBZ_ substitution is absorbed in |*F*/*C*_1*h*_| = *N*_***k***_/*N*_***k***_^IBZ^.

Therefore, for a given material, one must evaluate eq 18, 19, or 20 according to its magnetic point group, which requires computing eq 16 for the appropriate subgroup of *O*(3) and parametrizing the IBZ according to its space group since the lattice type determines the BZ. In List 1 below, we show, for each (unitary) non-centrosymmetric point group, the values of eq 16 in the simplified notation *aa*′;*bb*′;*cc*′, excluding the vanishing components as well as those that are redundant due to the permutation properties *aa*′;*bb*′;*cc*′ = *aa*′;*cc*′;*bb*′ = *bb*′;*aa*′;*cc*′ = *bb*′;*cc*′;*aa*′ = *cc*′;*aa*′;*bb*′ = *cc*′;*bb*′;*aa*′ and the reciprocity relation *a*′*a*;*b*′*b*;*c*′*c* = *aa*′;*bb*′;*cc*′. Afterward, the finite components of the σ_shift_^*a*;*bc*^ tensor and the relations between them are displayed, omitting the persistent  and specifying which of eq 18 or 19 is finite if only one of them is.^h^ When multiple orientations along the Cartesian axes are possible, we indicate the orientation of the generating operations with the notation *c*_*n*,*a*_ for a counterclockwise (2π/*n*)-fold rotation with axis along *a*, σ_*a*_ for a reflection through a plane perpendicular to *a*, and *s*_*n*,*a*_ for the improper rotation *c*_*n*,*a*_σ_*a*_. If a different orientation is employed, then there exists a transformation *r* ∈ *O*(3) of our coordinate system that matches the chosen orientation, and the new conductivity tensor  can be computed from the one given here as , which will often result in a simple permutation of coordinates.

On the other hand, the grid of the IBZ can be obtained (nonuniquely) as a restriction of a larger grid by imposing a set of linear constraints on the coefficients α_*i*_ of the  points (“dim” being the dimension of the lattice) in the basis of reciprocal lattice vectors ***G***_*i*_. Specifically
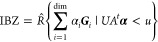
21where *U* and *u* depend on the space group and are given in List 1 for a particular choice of reciprocal lattice vectors ***G***_*i*_^ref^, which are those of ref (55) (see Tables 3.1 and 3.3 therein) and which we indicate in List 1 in 2π units for each lattice type under the first point group for which they appear. The coefficients α_*i*_ should initially be spanning the (−1, 1) interval because, while a range of length 1 for each would suffice, it is not guaranteed that our specific parametrization yields the same range for each coefficient^i^. In eq 21, *A* and *R* are introduced to allow for different sets of lattice vectors: *A* is the transformation matrix relating both sets of reciprocal vectors as
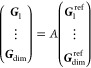
and *R̂* is the operator represented by the *R* ∈ *SO*(dim) rotation matrix that relates both BZs, acting on the set of ***k*** points to its right. The parametrized IBZs lie within the BZ except for the triclinic and monoclinic systems. The space groups *G* are labeled by their international number, and the different variations of IBZs that cannot be obtained through rotations are considered.

List 1 can also be employed with 2D materials in the following way: first identify the 3D space group that is compatible with the 2D structure, taking into account that in this case only groups with ***G***_3_^ref^∥*z* and lacking nonsymmorphic translations along *z* can be compatible. Then, if *F* = *C*_2_ or *C*_2*v*_, permute two columns of *U* to match the *c*_2_ axis orientation,^j^ if *F* = *C*_*s*_ (σ_*y*_) do the columns permutation (123) → (132), if *F* = *C*_*s*_ (σ_*x*_) do the columns permutation (123) → (231); and if any of these 3 permutations was performed, do the same permutation of the (*x*, *y*, *z*) indices in *aa*′;*bb*′;*cc*′; and σ^*a*;*bc*^. Finally, eliminate all rows in *U* and *u* whose first two entries in *U* are zero. The reduction of IBZs with  has been chosen such that the last step yields the correct IBZ in 2D (invariant with *c*_2,*z*_ or halved without it) with all compatible space groups.

Either the *italic* or the **bold** rows in *U* and *u* are included for a given system; in particular, the *italic* rows (excluding the **bold**) are included when the IBZ is not affected by , and the **bold** rows (excluding the *italic*) when the IBZ is halved by . This can be determined for the four types of magnetic space groups,^55^ which we denote as *MG*, as an application of the previous discussion of magnetic point groups, in addition to the specific formula for σ_shift_^*a*;*bc*^.

- Magnetic space group of type I: *MG* = *G* → *italic* rows, eq 18.
- Magnetic space group of type II: **bold** rows, eq 19.
- Magnetic space group of type III: , where *G̃* is a space group whose point group has half the order of *G*’s point group, the latter of which determines the IBZ → *italic* rows, eq 20.
- Magnetic space group of type IV: , where (*e*|***t***_0_) ∉ *G* is a pure translation. The IBZ is defined by *G*, for our purpose with  since the extra translation, as in nonsymmorphic groups, is inconsequential → **bold** rows, eq 19.

### List 1

Independent nonvanishing components of eq 16 and the σ_shift_^*a*;*bc*^ tensor, the relations between them, and the prescription for generating the IBZ for each non-centrosymmetric space group.

(a) *F* = *C*_1_
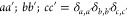

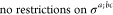

- *G* = 1 [Γ_*t*_]

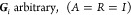

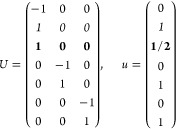


(b) *F* = *C*_2_ (*c*_2,*z*_)
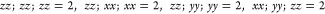

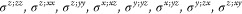

- *G* = 3, 4 [Γ_*m*_]

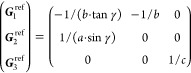

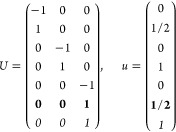

- *G* = 5 [Γ_*m*_^*b*^]

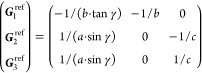

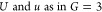


(c) *F* = *C*_*s*_ (σ_*z*_)
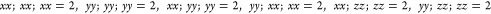

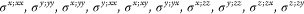

- *G* = 6, 7 [Γ_*m*_]

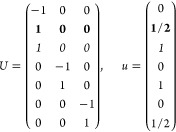

- *G* = 8, 9 [Γ_*m*_^*b*^]

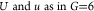


(d) *F* = *D*_2_
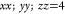

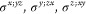

- *G* = 16–19 [Γ_*o*_]

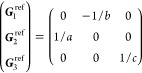

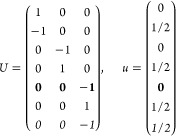

- *G* = 20, 21 [Γ_*o*_^*b*^].

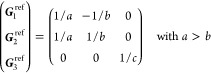

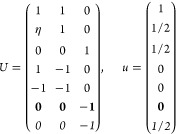

- *G* = 22 [Γ_*o*_^*f*^]

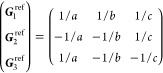

Variation 1: *a*^–2^ ≥ *b*^–2^ + *c*^–2^. ,

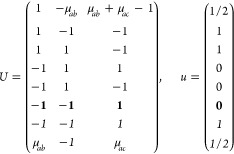

Variation 2: *a*^–2^ < *b*^–2^ + *c*^–2^ and *b*^–2^ < *c*^–2^ + *a*^–2^. ,

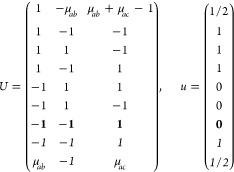

- *G* = 23, 24 [Γ_*o*_^*v*^]. , ,

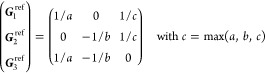

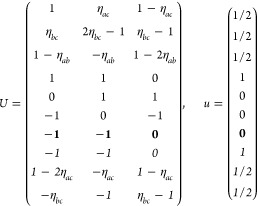


(e) *F* = *C*_2*v*_ (*c*_2,*z*_, σ_*x*_)
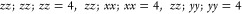

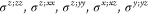

- *G* = 25–34 [Γ_*o*_] → Same as *G* = 16
- *G* = 35–41 [Γ_*o*_^*b*^] → Same as *G* = 20
- *G* = 42, 43 [Γ_*o*_^*f*^] → Same as *G* = 22
- *G* = 44–46 [Γ_*o*_^*v*^] → Same as *G* = 23

(f) *F* = *C*_4_ (*c*_4,*z*_)
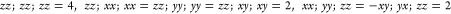

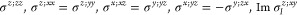

- *G* = 75–78 [Γ_*q*_]

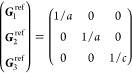

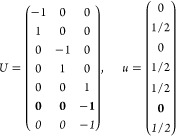

- *G* = 79, 80 [Γ_*q*_^*v*^]

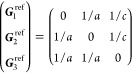

Variation 1: *a* > *c*.

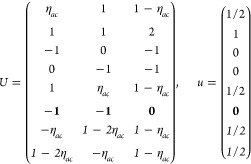

Variation 2: *c* > *a*.

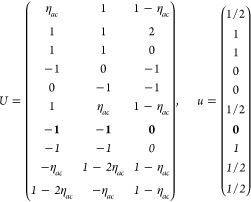


(g) *F* = *S*_4_ (*s*_4,*z*_)
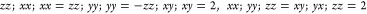

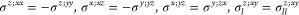

- *G* = 81 [Γ_*q*_] → Same as *G* = 75
- *G* = 82 [Γ_*q*_^*v*^] → Same as *G* = 79

(h) *F* = *D*_4_ (*c*_4,*z*_, *c*_2,*x*_)
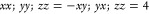

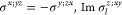

- *G* = 89–96 [Γ_*q*_]

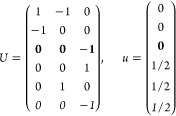

- *G* = 97, 98 [Γ_*q*_^*v*^]

Variation 1: *a* > *c*.

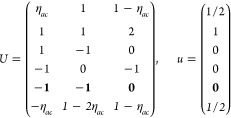

Variation 2: *c* > *a*.

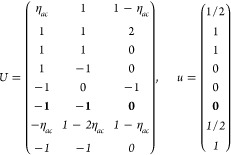


(i) *F* = *C*_4*v*_ (*c*_4,*z*_, σ_*x*_)
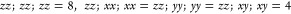

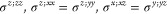

- *G* = 99–106 [Γ_*q*_] → Same as *G* = 89
- *G* = 107–110 [Γ_*q*_^*v*^] → Same as *G* = 97

(j) *F* = *D*_2*d*_ (*s*_4,*z*_, *c*_2,*x*_)
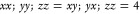

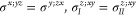

- *G* = 111–118 [Γ_*q*_] → Same as *G* = 89
- *G* = 119–122 [Γ_*q*_^*v*^] → Same as *G* = 97

(k) *F* = *C*_3_ (*c*_3,*z*_)
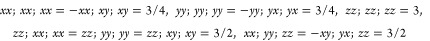

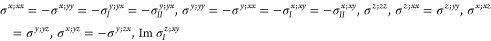

- *G* = 143–145 [Γ_*h*_]

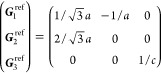

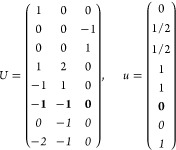

- *G* = 146 [Γ_*rh*_]

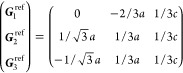

Variation 1:.

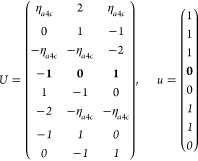

Variation 2:. ,

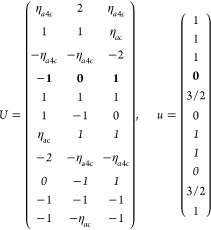


(l) *F* = *D*_3_ (*c*_3,*z*_, *c*_2,*x*_)
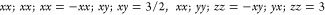

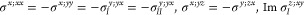

- *G* = 149–154 [Γ_*h*_]

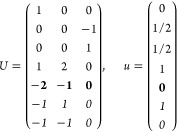

- *G* = 155 [Γ_*rh*_]

Variation 1:.

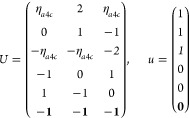

Variation 2:. ,

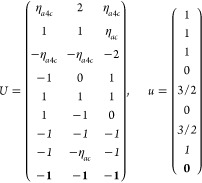


(m) *F* = *C*_3*v*_ (*c*_3,*z*_, σ_*y*_)
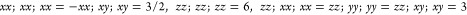

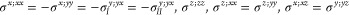

- *G* = 156–159 [Γ_*h*_] → Same as *G* = 149
- *G* = 160, 161 [Γ_*rh*_] → Same as *G* = 155

(n) *F* = *C*_6_ (*c*_6,*z*_)
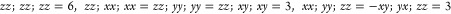

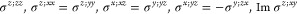

- *G* = 168–173 [Γ_*h*_]

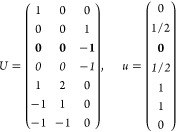


(o) *F* = *C*_3*h*_ (*c*_3,*z*_, σ_*z*_)
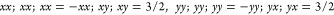

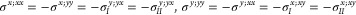

- *G* = 174 [Γ_*h*_]

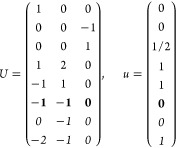


(p) *F* = *D*_6_ (*c*_6,*z*_, *c*_2,*x*_)
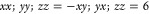

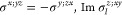

- *G* = 177–182 [Γ_*h*_]

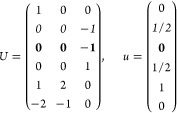


(q) *F* = *C*_6*v*_ (*c*_6,*z*_, σ_*x*_)
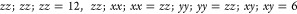

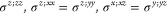

- *G* = 183–186 [Γ_*h*_] → Same as *G* = 177

(r) *F* = *D*_3*h*_ (*s*_3,*z*_, *c*_2,*x*_)
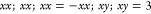

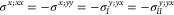

- *G* = 187–190 [Γ_*h*_]

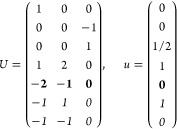


(s) *F* = *T*
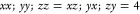

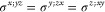

- *G* = 195, 198 [Γ_*c*_]

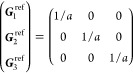

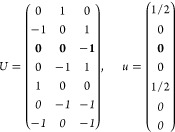

- *G* = 196 [Γ_*c*_^*f*^]

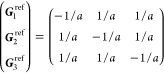

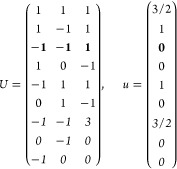

- *G* = 197, 199 [Γ_*c*_^*v*^]

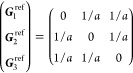

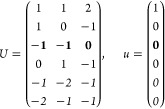


(t) *F* = *O*
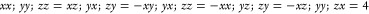

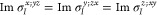

- *G* = 207, 208, 212, 213 [Γ_*c*_]

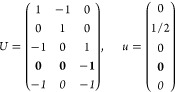

- *G* = 209, 210 [Γ_*c*_^*f*^]

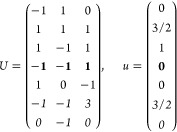

- *G* = 211, 214 [Γ_*c*_^*v*^]

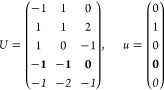


(u) *F* = *T*_*d*_
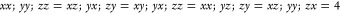

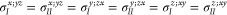

- *G* = 215, 218 [Γ_*c*_] → Same as *G* = 207
- *G* = 216, 219 [Γ_*c*_^*f*^] → Same as *G* = 209
- *G* = 217, 220 [Γ_*c*_^*v*^] → Same as *G* = 211

### Explicit Expressions in Type III Magnetic Groups

The folded equation for magnetic point groups of type III (eq 20) can be readily computed from List 1. Since the evaluation is slightly more involved, we next provide in List 2 the explicit expressions for all nonvanishing components σ_shift_^*a*;*bc*^ in the nontrivial cases, namely, for those groups *M* where *H* is non-centrosymmetric (*i* ∈ *H* ⇒ σ_shift_^*a*;*bc*^ = 0) or there is  symmetry , in which case eq 20 reduces to
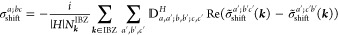
which can be evaluated straightforwardly as eq 18 or 19. The general form of the shift conductivity tensor can then be immediately identified from these expressions for any magnetic point group. The type III groups are labeled with an arbitrary number and in the Shubnikov–Belov notation as in ref (55). The orientation of each ordinary point group *H* and *F* is the same as in List 1, except when it is specified by new generators. For simplicity, we omit the “shift” label, the ***k*** dependence, and the ubiquitous  in the notation of the conductivity. The general relations  are also omitted.

### List 2

- M = 2 (2′), *H* = *C*_1_, *F* = *C*_2_

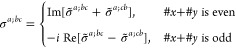


where *#x* (*#y*) is the number of times that *x* (*y*) appears in the (*a*, *b*, *c*) triplet.

- *M* = 3 (*m*′), *H* = *C*_1_, *F* = *C*_*s*_

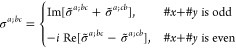


where *#x* (*#y*) is the number of times that *x* (*y*) appears in the (*a*, *b*, *c*) triplet.

- *M* = 7 (2′2′2), *H* = *C*_2_, *F* = *D*_2_

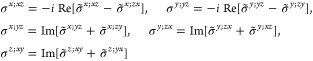

- *M* = 8 (*m*′*m*′2), *H* = *C*_2_, *F* = *C*_2*v*_

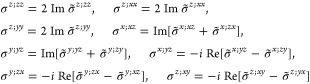

- *M* = 9 (*m*′*m*2′), *H* = *C*_*s*_ (σ_*y*_), *F* = *C*_2*v*_

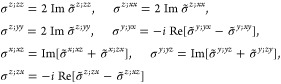

- *M* = 13 (4′), *H* = *C*_2_, *F* = *C*_4_

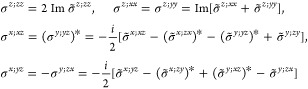

- *M* = 14 (4̅′), *H* = *C*_2_, *F* = *S*_4_

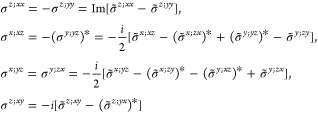

- *M* = 15 (42′2′), *H* = *C*_4_, *F* = *D*_4_

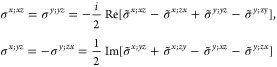

- *M* = 16 (4′22′), *H* = *D*_2_, *F* = *D*_4_

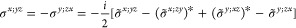

- *M* = 20 (4*m*′*m*′), *H* = *C*_4_, *F* = *C*_4*v*_

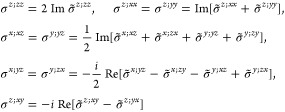

- *M* = 21 (4′*mm*′), *H* = *C*_2*v*_, *F* = *C*_4*v*_

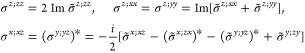

- *M* = 22 (4̅2′*m*′), *H* = *S*_4_, *F* = *D*_2*d*_

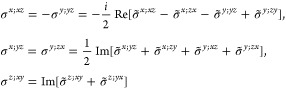

- *M* = 23 (4̅′2*m*′), *H* = *D*_2_, *F* = *D*_2*d*_

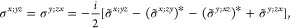

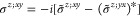

- *M* = 24 (4̅′*m*2′), *H* = *C*_2*v*_, *F* = *D*_2*d*_ (*s*_4,*z*_, σ_*x*_)

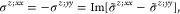

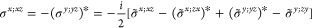

- *M* = 30 (32′), *H* = *C*_3_, *F* = *D*_3_

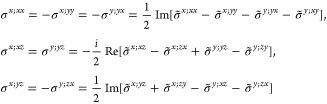

- *M* = 31 (3*m*′), *H* = *C*_3_, *F* = *C*_3*v*_

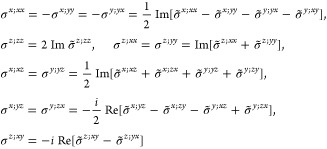

- *M* = 32 (6̅′), *H* = *C*_3_, *F* = *C*_3*h*_

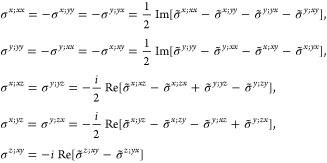

- *M* = 33 (6̅*m*′2′), *H* = *C*_3*h*_, *F* = *D*_3*h*_

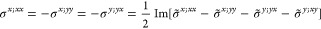

- *M* = 34 (6̅′*m*2′), *H* = *C*_3*v*_, *F* = *D*_3*h*_

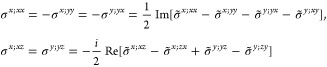

- *M* = 35 (6̅′*m*′2), *H* = *D*_3_, *F* = *D*_3*h*_

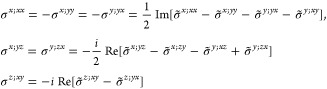

- *M* = 36 (6′), *H* = *C*_3_, *F* = *C*_6_

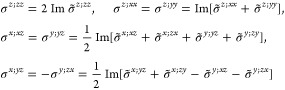

- *M* = 41 (62′2′), *H* = *C*_6_, *F* = *D*_6_

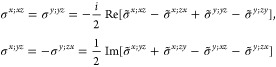

- *M* = 42 (6′2′2), *H* = *D*_3_, *F* = *D*_6_

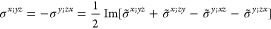

- *M* = 46 (6*m*′*m*′), *H* = *C*_6_, *F* = *C*_6*v*_

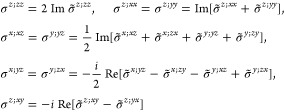

- *M* = 47 (6′*m*′*m*), *H* = *C*_3*v*_, *F* = *C*_6*v*_

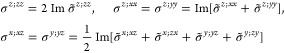

- *M* = 54 (4̅′3*m*′), *H* = *T*, *F* = *T*_*d*_

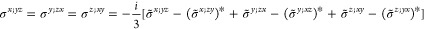

- *M* = 55 (4′32′), *H* = *T*, *F* = *O*

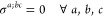


## 4 Conclusive Remarks

The use of Gaussian basis sets to compute the shift conductivity has been proven satisfactory. The analytical evaluation of real-space integrals involving localized functions allows to readily compute the Berry connection and velocity matrix elements, while the reduced dimension yields lighter calculations, and the economical option of hybrid functionals allows to easily reproduce the desired band gap with good precision in a wide variety of systems. Furthermore, the (magnetic) space group symmetry is fully preserved, and it can be capitalized on to perform maximally efficient reciprocal space summations, in addition to immediately discerning the contributions to the electrical current under any light polarization.

The numerical results for the chosen materials are in standard agreement with the literature and, in all cases, the length and velocity gauges have been shown to yield nearly identical outcomes. One would be tempted to conclude that the velocity gauge, in view of its comparative simplicity, should then be the preferred option in general. However, the length gauge makes no use of completeness relations, and larger discrepancies may appear when employing smaller bases. In addition, care should be taken when separating the shift and injection contributions without time-reversal symmetry in the velocity gauge. Nevertheless, the use of as-largest-as-possible basis sets is generally advisable, typically between TZVP and QZVP and including diffuse exponents. The use of ghost atoms, i.e., basis functions not located on atomic positions, could be explored in order to facilitate the reproduction of particularly delocalized empty conduction states.

We note that all other single-particle contributions to the BPVE (in particular, the injection current) and to the total second-order optical response (in particular, the second-harmonic generation) can be computed from this method, since the corresponding expressions involve the same basic quantities as the shift current. The transformation properties of these other optical contributions are the same for spatial operations, but the role of time-reversal may change. For example, the injection conductivity has the opposite behavior to σ_shift_, in the sense that time-reversal symmetry forces it to be imaginary (instead of real); and the results in Section 3 can be adapted from that. The evaluation of metallic systems is also possible in the length gauge, albeit it introduces additional terms with Fermi surface derivatives.^33^

## Supporting Information Available

Input files for the self-consistent electronic structure calculations in CRYSTAL23 for each material; band structures along high-symmetry lines for each material; and explicit comparison between the BZ and IBZ summations in BaTiO_3_ (PDF)

## Footnotes

a Which, in practice, is not an actual (complete) basis in this function space due to its finiteness. Nevertheless, we use this term to refer to noncomplete sets according to standard convention.

b In charge-neutral 2D systems, the Coulomb potential may be replaced by the Parry potential instead of the usual Ewald potential in 3D.^40^

c Ghost atoms would be treated analogously as long as they preserved the space group symmetry.

d The last identity results from the expansion of

e The grid choices here were influenced by benchmarking purposes, and substantially coarser ones will often yield good results.

f Note that the translations commute with *Ĥ* as fixed parameters.

g The space group can be regarded as unaltered if projective representations of the little groups are employed,^44^ which do not appear in this derivation regardless. Alternatively, vector (standard) representations of enlarged little groups can be considered.^55^ On the other hand, we disregard the extra spin-only operations of the spin-space group that may appear without SOC. These may possibly introduce further restrictions, but they would not overrule the present ones in any case.

h For example, Im σ_I_^*x*;*yz*^ indicates that (eq 18) is purely imaginary and (eq 19) is null for the *a* = *x*, *b* = *y*, and *c* = *z* component. Note that σ_I_^*a*;*bc*^ = σ_II_^*a*;*bc*^ implies that (eq 18) is real. We omit the mandatory equalities between (eqs 18 and 19) for *b* = *c* components.

i Note that, in this case, a *N*_1_ × *N*_2_ × *N*_3_ grid for the {α_1_,α_2_,α_3_} coefficients corresponds to a (*N*_1_/2) × (*N*_2_/2) × (*N*_3_/2) grid of the BZ.

j For example, in the GeS *C*_2*v*_ (*c*_2*x*_) geometry of the present work, the first and third columns are permuted.
