# Supplementary material for: Shift Current with Gaussian Basis Sets and General Prescription for Maximally Symmetric Summations in the Irreducible Brillouin Zone
Source: J Chem Theory Comput. 2023 Dec 14;19(24):9416–34. doi: 10.1021/acs.jctc.3c00917 (PMC10753807; doi:10.1021/acs.jctc.3c00917)
Supplement: Supplementary file 1 — ct3c00917_si_001.pdf [file ct3c00917_si_001.pdf]

Shift current with Gaussian basis sets & general prescription for maximally-symmetric summations in the irreducible Brillouin zone - SI

M. A. García-Blázquez,*,† J. J. Esteve-Paredes,† A. J. Uría,† and J. J. Palacios†,‡

† *Departamento de Física de la Materia Condensada, Universidad Autónoma de Madrid, E-28049 Madrid, Spain*

‡*Condensed Matter Physics Center (IFIMAC), Universidad Autónoma de Madrid, E-28049 Madrid, Spain*

E-mail: manuelantonio.garcia@estudiante.uam.es

1 CRYSTAL input files & band structures

1.1 MoS_2

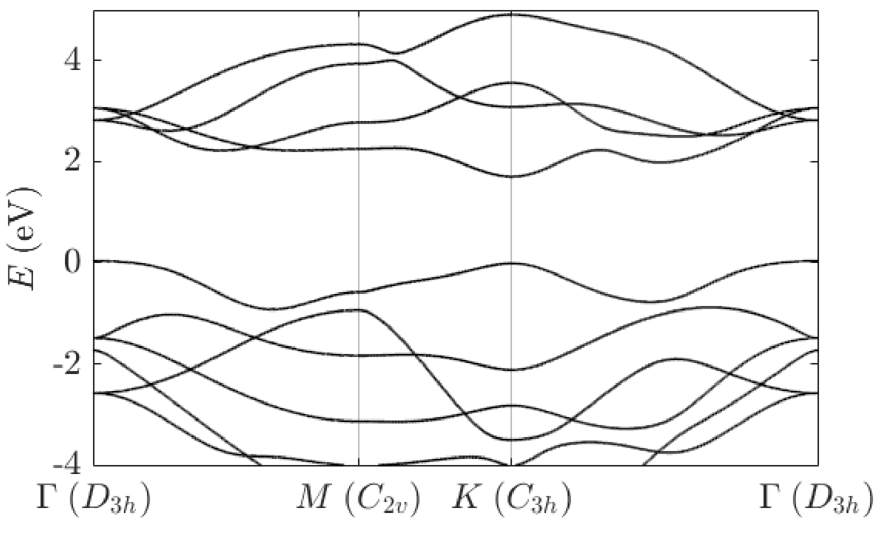

```
SLAB
69
3.184
3
16 0.66666666667 0.33333333333 1.56355632
16 0.66666666667 0.33333333333 -1.56355632
242 0.33333333333 0.66666666667 0.
END
16 19
0 0 10 2.0 1.0
1273410.9023000 0.11767088246D-04
190697.8300700 0.91478610166D-04
43397.8853300 0.48090078640D-03
12291.8096770 0.20257193592D-02
4009.7420824 0.73190096406D-02
1447.3531030 0.23300499900D-01
564.30102913 0.65386213610D-01
233.74506243 0.15614449910
101.56402814 0.29318563787
45.805907187 0.36287914289
0 0 3 2.0 1.0
394.27281503 0.18753305081D-01
121.72249591 0.16870726663
46.754125963 0.63806830653
0 0 1 2.0 1.0
20.923008254 1.0000000
0 0 1 0.0 1.0
```

8.2685567800 1.0000000

0 0 1 0.0 1.0

3.8629345671 1.0000000

0 0 1 0.0 1.0

1.7794684781 1.0000000

0 0 1 0.0 1.0

0.61064260103 1.0000000

0 0 1 0.0 1.0

0.27412269445 1.0000000

0 0 1 0.0 1.0

0.11325939107 1.0000000

0 2 8 6.0 1.0

2189.8930459 0.23912552864D-03

518.94596592 0.20772032158D-02

168.19560151 0.11242420571D-01

63.745282788 0.44069933941D-01

26.597033077 0.12918778608

11.774251449 0.26910820167

5.3534379024 0.37855928620

2.4701911802 0.29692134655

0 2 2 4.0 1.0

82.120288349 -0.39420318847D-01

4.9523532869 0.64048403090

0 2 1 0.0 1.0

1.0828262029 1.0000000

0 2 1 0.0 1.0

0.49271277356 1.0000000

```
0 2 1 0.0 1.0
0.20483450942 1.0000000
0 2 1 0.0 1.0
0.80743615716D-01 1.0000000
0 3 1 0.0 1.0
4.15900000 1.0000000
0 3 1 0.0 1.0
1.01900000 1.0000000
0 3 1 0.0 1.0
0.464000000 1.0000000
0 3 1 0.0 1.0
0.194000000 1.0000000
242 12
INPUT
14.  0 2 4 4 2 0
10.097000 180.076853 0
4.375670 24.715920 0
9.126564 41.227678 0
8.863223 82.452670 0
4.044948 6.345092 0
3.866657 12.458423 0
7.535754 19.308744 0
7.278976 28.977674 0
2.763205 3.189516 0
2.772085 4.700169 0
6.306633 -7.178888 0
6.356448 -9.745978 0
```

```
0 0 2 2 1.0
14.0000000000 -0.224900434060
12.5000000000 0.331512485550
0 0 1 2 1.0
4.25053415000 1.000000000000
0 0 1 0 1.0
0.65151308000 1.000000000000
0 0 1 0 1.0
0.18122290000 1.000000000000
0 2 4 6 1.0
8.89311179150 0.069994449475
5.46891122700 -0.235471418830
1.35484730070 0.463154600070
0.65494867461 0.488201847100
0 2 1 0 1.0
0.46348506000 1.000000000000
0 2 1 0 1.0
0.24987406000 1.000000000000
0 2 1 0 1.0
0.1 1.000000000000
0 3 3 4 1.0
5.00444454970 -0.021587364862
1.77368233240 0.209586800860
0.76950591696 0.437308805990
0 3 1 0 1.0
0.56023361000 1.000000000000
0 3 1 0 1.0
```

```
0.20486424000 1.000000000000

0 4 1 0 1.0

0.55911598000 1.000000000000

99 0

PRINT

END

DFT

XXLGRID

PBEXC

ENDDFT

TOLINTEG

10 10 10 10 30

SHRINK

24 24 24

MAXCYCLE

300

FMIXING

90

TOLDEE

12

NODIIS

END
```

1.2 GeS

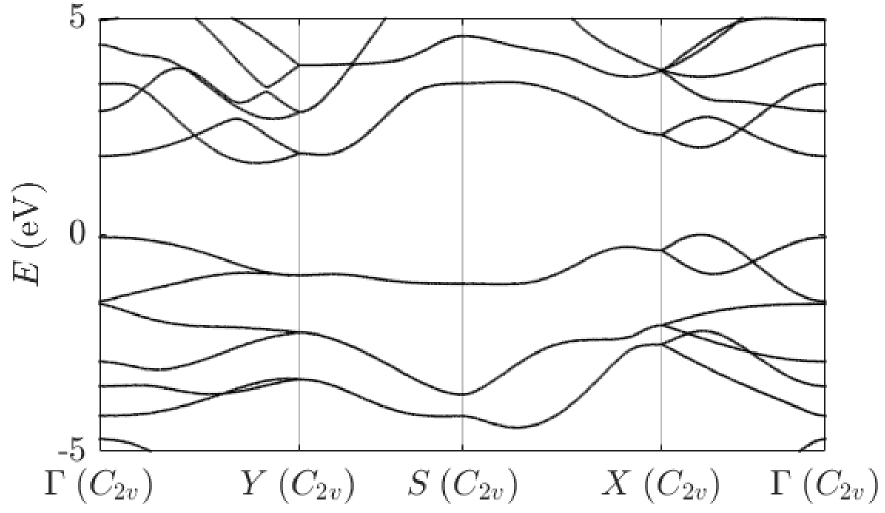


```
SLAB

31

4.47 3.66

2

16 0.000000 0.000000 1.07

32 -0.131991 0.000000 -1.28

END

16 19

0 0 10 2.0 1.0

1273410.9023000 0.11767088246D-04

190697.8300700 0.91478610166D-04

43397.8853300 0.48090078640D-03

12291.8096770 0.20257193592D-02

4009.7420824 0.73190096406D-02

1447.3531030 0.23300499900D-01

564.30102913 0.65386213610D-01

233.74506243 0.15614449910
```

101.56402814 0.29318563787

45.805907187 0.36287914289

0 0 3 2.0 1.0

394.27281503 0.18753305081D-01

121.72249591 0.16870726663

46.754125963 0.63806830653

0 0 1 2.0 1.0

20.923008254 1.0000000

0 0 1 0.0 1.0

8.2685567800 1.0000000

0 0 1 0.0 1.0

3.8629345671 1.0000000

0 0 1 0.0 1.0

1.7794684781 1.0000000

0 0 1 0.0 1.0

0.61064260103 1.0000000

0 0 1 0.0 1.0

0.27412269445 1.0000000

0 0 1 0.0 1.0

0.11325939107 1.0000000

0 2 8 6.0 1.0

2189.8930459 0.23912552864D-03

518.94596592 0.20772032158D-02

168.19560151 0.11242420571D-01

63.745282788 0.44069933941D-01

26.597033077 0.12918778608

11.774251449 0.26910820167

5.3534379024 0.37855928620

2.4701911802 0.29692134655

0 2 2 4.0 1.0

82.120288349 -0.39420318847D-01

4.9523532869 0.64048403090

0 2 1 0.0 1.0

1.0828262029 1.0000000

0 2 1 0.0 1.0

0.49271277356 1.0000000

0 2 1 0.0 1.0

0.20483450942 1.0000000

0 2 1 0.0 1.0

0.80743615716D-01 1.0000000

0 3 1 0.0 1.0

4.15900000 1.0000000

0 3 1 0.0 1.0

1.01900000 1.0000000

0 3 1 0.0 1.0

0.464000000 1.0000000

0 3 1 0.0 1.0

0.194000000 1.0000000

32 22

0 0 11 2.0 1.0

7233056.0346000 0.76638751457D-05

1082886.1731000 0.59603601369D-04

246481.4695900 0.31319088031D-03

69862.4269550 0.13194051561D-02

22815.8096620 0.47736099191D-02

8246.5369297 0.15319250467D-01

3219.9367257 0.43900870673D-01

1336.5743706 0.11028573632

582.87737501 0.22912630489

264.59511360 0.34779259246

123.77823320 0.29968722223

0 0 4 2.0 1.0

2311.1055804 0.75033084731D-02

716.27089868 0.74778386626D-01

275.45330910 0.35092882302

118.93292565 0.72055989686

0 0 1 2.0 1.0

58.435699085 1.0000000

0 0 1 2.0 1.0

26.261575973 1.0000000

0 0 1 0.0 1.0

12.664880671 1.0000000

0 0 1 0.0 1.0

5.7269548505 1.0000000

0 0 1 0.0 1.0

2.7555023203 1.0000000

0 0 1 0.0 1.0

1.2432886754 1.0000000

0 0 1 0.0 1.0

0.33107183846 1.0000000

0 0 1 0.0 1.0

0.15964081368 1.0000000

0 0 1 0.0 1.0

0.68463178923D-01 1.0000000

0 2 9 6.0 1.0

16555.7110740 0.10199370391D-03

3914.9903745 0.90504190546D-03

1269.4766518 0.51558490762D-02

484.35437789 0.22010335708D-01

204.64985822 0.73434798827D-01

92.791032094 0.18608955294

44.055060255 0.33330981945

21.601319931 0.36229776246

10.732169233 0.17765186263

0 2 2 6.0 1.0

100.88933962 -0.11146182144

36.640254741 -0.52324324314

0 2 4 2.0 1.0

8.0343224115 0.31484763263

4.5613438333 0.38737744790

3.1794882223 0.50034353638

0.92612041457 0.22656707948

0 2 1 0.0 1.0

1.7977040907 1.0000000

0 2 1 0.0 1.0

0.41386865432 1.0000000

0 2 1 0.0 1.0

0.15830798709 1.0000000

```
0 2 1 0.0 1.0
0.57877035911D-01 1.0000000
0 3 7 10.0 1.0
420.99565921 0.78212192196D-03
126.36209818 0.69560123831D-02
48.661473548 0.33712188323D-01
20.880325527 0.10717291194
9.5418229355 0.23161030141
4.4353653868 0.33390954301
2.0285421942 0.33720676378
0 3 1 0.0 1.0
0.89484935267 0.21702473265
0 3 1 0.0 1.0
0.35786074334 1.0000000
0 3 1 0.0 1.0
0.1400000 1.0000000
99 0
PRINT
END
DFT
XXLGRID
PBEXC
END
TOLINTEG
10 10 10 10 30
SHRINK
24 24 24
```

```
MAXCYCLE

300

FMIXING

40

TOLDEE

12

END
```

1.3 GaAs

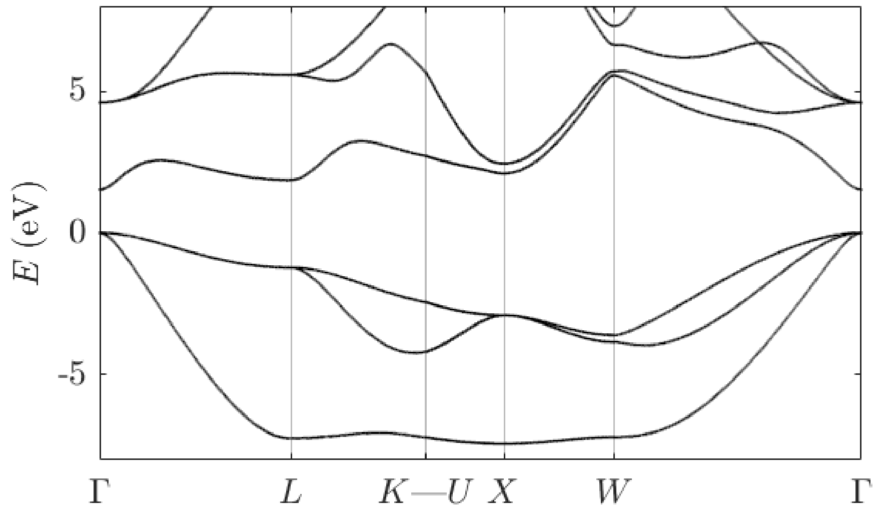


```
CRYSTAL
0 0 0
216
5.65
2
231 0.  0.  0.
233 0.25 0.75 0.75
END
231 9
INPUT
21.  0 2 4 6 2 0
25.880361 370.273040 0
7.901295 9.190615 0
45.149190 99.144001 0
44.979981 198.295512 0
17.224251 28.445653 0
16.747329 56.949705 0
```

```
51.968812 -18.168797 0
51.629117 -27.380273 0
15.241738 -1.587022 0
15.320193 -2.516292 0
4.918589 0.083166 0
4.755103 0.202198 0
10.762263 -0.616990 0
19.852939 -3.138584 0
0 0 6 2.  1.
2848.20000 0.362000000E-03
420.664000 0.211700000E-02
29.8118000 0.118964000
14.2207000 -0.461723000
2.67643000 0.751559000
1.13353000 0.447202000
0 0 6 2.  1.
2848.20000 -0.970000000E-04
420.664000 -0.614000000E-03
29.8118000 -0.310690000E-01
14.2207000 0.126784000
2.67643000 -0.264288000
1.13353000 -0.275471000
0 0 1 0.  1.
0.207220000 1.00000000
0 0 1 0.  1.
0.120000000 1.00000000
0 2 6 6.  1.
```

```
109.624000 0.210100000E-02
21.0855000 -0.801960000E-01
4.92260000 0.396415000
2.15591000 0.519076000
0.901913000 0.207520000
0.202004000 0.782500000E-02
0 2 6 1.  1.
109.624000 -0.288000000E-03
21.0855000 0.135550000E-01
4.92260000 -0.736290000E-01
2.15591000 -0.120860000
0.901913000 -0.196000000E-02
0.202004000 0.493206000
0 2 1 0.  1.
0.120000000 1.00000000
0 3 6 10.  1.
85.7978000 0.146680000E-01
27.6822000 0.856210000E-01
10.1760000 0.248336000
3.92208000 0.401414000
1.45858000 0.398604000
0.488760000 0.186898000
0 3 1 0.  1.
0.177200000 1.00000000
233 9
INPUT
23.  0 2 4 6 2 0
```

28.725122 370.114025 0

6.767681 9.349296 0

45.331064 99.142103 0

44.767415 198.307880 0

19.539090 28.383073 0

18.973471 56.871464 0

51.057152 -18.485145 0

50.151340 -28.113530 0

16.108936 -1.223895 0

14.672223 -1.345765 0

3.851927 0.101757 0

3.813502 0.170338 0

11.940584 -0.775230 0

17.761160 -2.157259 0

0 0 6 2. 1.

2542.81000 0.113700000E-02

381.169000 0.605500000E-02

40.2342000 0.841250000E-01

16.1217000 -0.405285000

3.20189000 0.712926000

1.42096000 0.473376000

0 0 6 2. 1.

2542.81000 -0.390000000E-03

381.169000 -0.219000000E-02

40.2342000 -0.268530000E-01

16.1217000 0.136878000

3.20189000 -0.320457000

1.42096000 -0.337391000

0 0 1 0. 1.

0.321443000 1.00000000

0 0 1 0. 1.

0.120000000 1.00000000

0 2 6 6. 1.

99.5349000 0.385700000E-02

24.1195000 -0.851010000E-01

5.84196000 0.404762000

2.56010000 0.531478000

1.09308000 0.184012000

0.318424000 0.576400000E-02

0 2 6 3. 1.

99.5349000 -0.772000000E-03

24.1195000 0.199410000E-01

5.84196000 -0.107210000

2.56010000 -0.172259000

1.09308000 0.876100000E-02

0.318424000 0.569744000

0 2 1 0. 1.

0.120000000 1.00000000

0 3 6 10. 1.

113.509000 0.119800000E-01

36.8872000 0.795440000E-01

13.6893000 0.236755000

5.38964000 0.401534000

2.08046000 0.406686000

```
0.737568000 0.173162000

0 3 1 0.  1.

0.307800000 1.00000000

99 0

PRINT

END

DFT

XXLGRID

HSE06

END

TOLINTEG

10 10 10 10 30

SHRINK

24 24 24

MAXCYCLE

300

FMIXING

40

TOLDEE

12

END
```

1.4 $BaTiO_3$

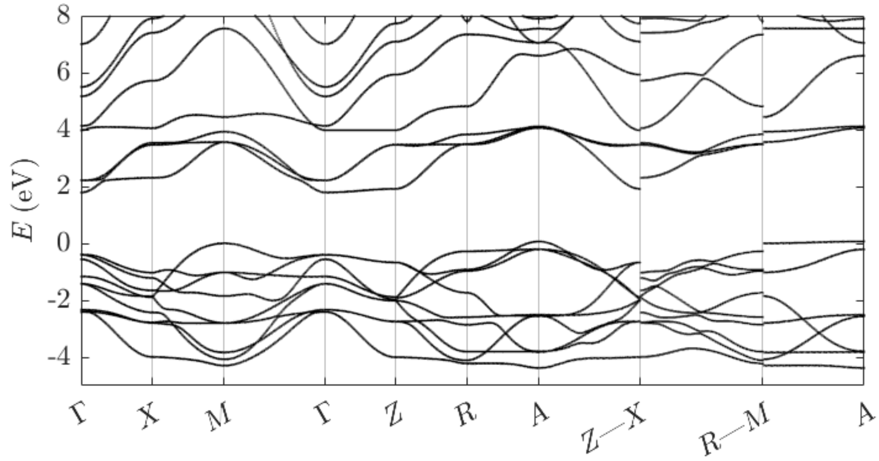


```
CRYSTAL
0 0 0
99
3.99 4.1
4
8 0.  0.  0.548972
8 0.  0.5 0.064221
22 0.  0.  0.100043
256 0.5 0.5 0.582541
END
8 12
0 0 8 2.0 1.0 116506.4690800 0.40383857939D-04
17504.3497240 0.31255139004D-03
3993.4513230 0.16341473495D-02
1133.0063186 0.68283224757D-02
369.99569594 0.24124410221D-01
133.62074349 0.72730206154D-01
52.035643649 0.17934429892
```

```
21.461939313 0.33059588895
0 0 2 2.0 1.0
89.835051252 0.96468652996D-01
26.428010844 0.94117481120
0 0 1 0.0 1.0
9.2822824649 1.0000000
0 0 1 0.0 1.0
4.0947728533 1.0000000
0 0 1 0.0 1.0
1.3255349078 1.0000000
0 0 1 0.0 1.0
0.51877230787 1.0000000
0 0 1 0.0 1.0
0.19772676454 1.0000000
0 2 5 4.0 1.0
191.15255810 0.25115697705D-02
45.233356739 0.20039240864D-01
14.353465922 0.93609064762D-01
5.2422371832 0.30618127124
2.0792418599 0.67810501439
0 2 1 0.0 1.0
0.84282371424 1.0000000
0 2 1 0.0 1.0
0.33617694891 1.0000000
0 2 1 0.0 1.0
0.12863997974 1.0000000
0 3 1 0.0 1.0
```

0.4534621300 1.0000000000000

22 18

0 0 11 2.0 1.0

3070548.8651000 0.86954016630D-05

460777.8864300 0.67452737727D-04

104901.2288900 0.35477293028D-03

29695.8611990 0.14977525588D-02

9678.8892688 0.54309912055D-02

3490.1877912 0.17439360524D-01

1359.2217621 0.49835634640D-01

562.42721208 0.12379633943

244.22296250 0.25057490943

110.16668710 0.35934609007

50.881903357 0.27594242664

0 0 4 2.0 1.0

965.95430789 0.41773927781D-02

299.27072059 0.40277148567D-01

114.83772939 0.17898686817

49.477578954 0.31783043543

0 0 1 2.0 1.0

22.982839977 1.0000000

0 0 1 2.0 1.0

10.518305037 1.0000000

0 0 1 0.0 1.0

4.9774390567 1.0000000

0 0 1 0.0 1.0

2.1339846838 1.0000000

0 0 1 0.0 1.0

1.0342457284 1.0000000

0 0 1 0.0 1.0

0.46199774995 1.0000000

0 0 1 0.0 1.0

0.10621264194 1.0000000

0 2 9 6.0 1.0

5169.6755427 0.18802523596D-03

1225.0961638 0.16473458826D-02

397.60051934 0.91104554321D-02

151.36154684 0.36987450926D-01

63.613321773 0.11376329624

28.514560307 0.25345208574

13.248003298 0.38019402704

6.3048807760 0.30989136346

2.9493525821 0.87418944007D-01

0 2 5 6.0 1.0

40.738772213 -0.73233793267D-02

14.062358461 -0.34282591082D-01

2.7460680961 0.35655009250

1.2713688141 0.76112035427

0.57610015545 0.62716743237

0 2 1 0.0 1

0.23981820724 1.0000000

0 2 1 0.0 1.0

0.11000000000 1.0000000

0 3 5 2.0 1.0

```
89.589880075 0.21223030030D-02
26.591412960 0.15911819913D-01
9.7739715702 0.62875243121D-01
3.9625083655 0.17144170807
1.6890532654 0.30565506624
0 3 1 0.0 1.0
0.71539771469 1.0000000
0 3 1 0.0 1.0
0.29366677748 1.0000000
0 3 1 0.0 1.0
0.11079094851 1.0000000
0 4 1 0.0 1.0
0.56200000000 1.00000000000000
256 10
INPUT
10.  0 2 4 4 4 2
4.177931587 84.785457583 0
2.522632800 17.372709041 0
6.294119351 52.512225743 0
6.476457746 105.022668647 0
2.284326647 8.707014937 0
2.091555201 17.165458832 0
1.925291745 5.346535679 0
1.878534118 8.025720742 0
0.907088727 1.346295081 0
0.910060953 2.063710453 0
6.256321669 -20.003223472 0
```

```
6.134135837 -26.118214748 0

1.641382784 -2.344457989 0

1.599343316 -2.980867480 0

2.142381001 -3.316602759 0

2.159981109 -4.275647018 0

0 0 2 2 1.0

5.7000000000 1.3292982002

5.2043612469 -1.7367497134

0 0 1 2 1.0

1.955486070000 1.000000000000

0 0 1 0 1.0

0.41033660537 1.0000000

0 0 1 0 1.0

0.18393532770 1.0000000

0 0 1 0 1.0

0.72521216545D-01 1.0000000

0 2 4 6 1.0

5.6000000000 0.51385026013

5.1104879274 -0.70529238864

2.5006544803 0.42822329830

0.51947995345 -0.57125984472

0 2 1 0 1.0

0.24536994841 1.0000000

0 2 1 0 1.0

0.11256056700 1.0000000

0 3 3 0 1.0

2.7000000000 .14372069823827
```

```
2.3460740881 -.19565347309413

0.39553542609 .29963058551591

0 3 1 0 1.0

0.12967935859 1.0000000

99 0

PRINT

END

DFT

XXLGRID

PBEXC

END

TOLINTEG

8 8 8 10 20

SHRINK

24 24 24

MAXCYCLE

500

FMIXING

95

TOLDEE

12

GUESSP

END
```

Note: the calculation was restarted from a previous iteration with MAXCYCLE= 500 and FMIXING= 90.

2 Comparison of BZ & IBZ summations

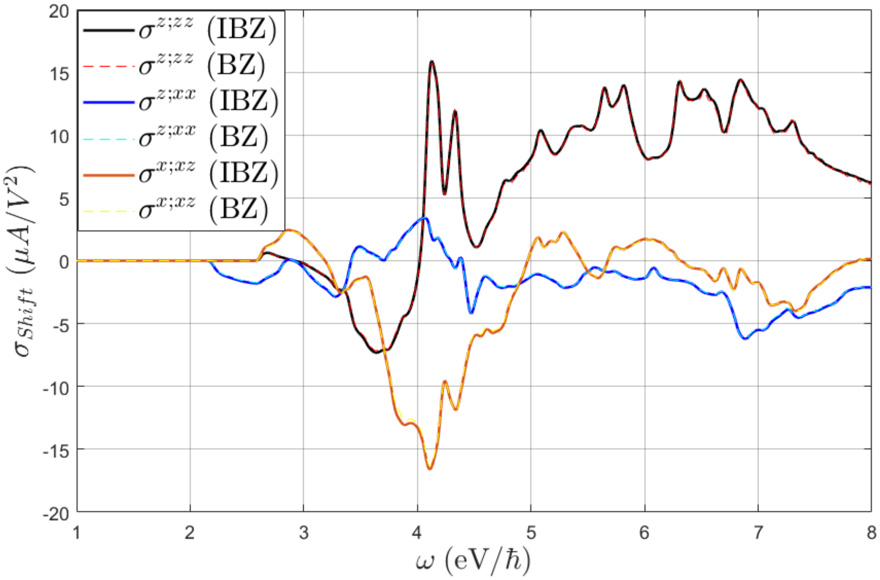

Figure 1: Shift conductivity tensor in $BaTiO_3$ computed in the velocity gauge with the standard BZ (dashed lines) and folded IBZ summations with time-reversal symmetry (solid lines). The BZ grid dimension was $100 \times 100 \times 100$, from which the IBZ grid was obtained by restriction as specified in the main text (other computational parameters as therein). The computation time ratio t_{BZ}/t_{IBZ} between the Brillouin zone (BZ) and irreducible Brillouin zone (IBZ, or representation domain) summations in identical computational conditions was 15.94, which is approximately Volume BZ/Volume IBZ $= 2|F| = |C_{4v} + \mathcal{T}C_{4v}| = 16$.
